# Antibody responses to intradermal or intramuscular MF59-adjuvanted influenza vaccines as evaluated in elderly institutionalized volunteers during a season of partial mismatching between vaccine and circulating A(H3N2) strains

**DOI:** 10.1186/1742-4933-11-10

**Published:** 2014-05-16

**Authors:** Barbara Camilloni, Michela Basileo, Angela Di Martino, Isabella Donatelli, Anna Maria Iorio

**Affiliations:** 1 Department Experimental Medicine, University of Perugia, Piazza Gambuli, Perugia 06132, Italy; 2 Department MIPI Istituto Superiore di Sanità, Viale Regina Elena 299, Rome 00161, Italy

**Keywords:** MF59-adjuvanted influenza vaccine, Intradermal influenza vaccine, Homologous and heterologous antibodies

## Abstract

**Background:**

The age-related weakening of the immune system makes elderly subjects less responsive to influenza vaccination. In the last years, two “enhanced vaccines” were licensed for individuals aged ≥65 years, one being a subunit vaccine (Fluad®) containing the MF59 adjuvant administered intramuscularly (IM-MF59) and the other one a split non-adjuvanted vaccine administered intradermally (Intanza® 15mcg) (ID). In the present study, we evaluated and compared the antibody responses against the three vaccine antigens and heterovariant A(H3N2) circulating viruses induced by IM-MF59 and ID influenza vaccines in 80 elderly institutionalized volunteers (40 per group) during the Winter season 2011–2012.

**Results:**

Hemagglutination inhibiting (HI) antibody titers were assessed in blood samples collected before, 1 and 6 months after vaccination. One month after vaccination both the IM-MF59 and ID vaccines induced increases in HI titers against all the three vaccine strains. The results in the two groups were similar against the A(H3N2) and A(H1N1) strains. Responses against the B strain typically tended to be higher after ID than IM-MF59, yet both vaccines stimulated lower responses against the B strain than against the two A strains. The two vaccines induced favorable results also against four epidemic drifted A(H3N2) viruses circulating in Winter 2011–2012. Six months after vaccination, the HI titers decreased in both groups.

**Conclusion:**

The responses induced by IM-MF59 and ID vaccines in institutionalized elderly people were similar against the A(H3N2) and A(H1N1) strains but frequently higher, for the ID, against the B strain. The two vaccines induced positive responses against drifted A(H3N2) circulating viruses.

## Background

Vaccination is the primary means of preventing seasonal influenza infection. However, although it can effectively prevent influenza and its complication in healthy adults, the age-related weakening of the immune system (immunosenescence) makes elderly subjects not only more susceptible to infection, but also less responsive to vaccination [1-4]. To meet the challenge of improving vaccine efficacy in the elderly and in other influenza risk groups, several strategies have been pursued [5]. Some of these research approaches have led to the licensure of new “enhanced vaccines” and two of these were specially licensed for individuals aged ≥65 years. The first one was a subunit vaccine containing the MF59 adjuvant (Fluad®) with the aim to increase vaccine immunogenicity, to be administered intramuscularly (IM-MF59) [6]. The second vaccine was a split non-adjuvanted vaccine administered intradermally (Intanza®) (ID), supposed to reach the same goal by reliably delivering the vaccine into the immune-rich environment of the dermis [7]. In most instances, both vaccines were found to be capable of inducing higher, or comparable, immune responses in the elderly when compared to conventional non-enhanced influenza vaccines [8-12]. Moreover, since mismatches between the vaccine strains and the circulating viruses can cause an additional reduction in vaccine efficacy [13], the two vaccines were also investigated, with favorable results, for their ability to induce antibody in the elderly not only against the vaccine strains, but also against heterovariant influenza strains [14]. The aim of our study was to evaluate and directly compare the ability of the two licensed enhanced vaccines to elicit an antibody response against the vaccine antigens. Moreover, we studied the long-term immunogenicity and the cross-responses against circulating mismatched influenza A(H3N2) viruses. The volunteers were institutionalized elderly people and the period of observation was the 2011–2012 Winter season.

## Results

### Characteristics of the study subjects

The study included a total of 80 elderly subjects living in two nursing homes located in Umbria, a region of central Italy, (42 at the “Opera Pia Bartolomei-Castori” and 38 at the “Casa Serena” nursing homes). Forty volunteers were vaccinated with IM-MF59 and 40 with ID influenza vaccine commercially available for the 2011–2012 Winter season.

As reported in Table 1, the baseline characteristics of the two groups were similar and for this reason the results obtained with the two vaccines could be compared.

**Table 1:** Baseline characteristics of elderly institutionalized subjects participating in the study

	**IM-MF59 (N. 40)**	**ID (N. 40)**	**p value**
% Female	87.5	82.5	0.745
Mean age (range)	85.3 (72–98)	84.6 (64–100)	0.683
% previous influenza vaccinated people *	100	100	1.000
% Underlying disease**	100	100	1.000
% Cardiovascular diseases	42.5	38.7	0.747
% Diabetes	15.0	22.6	0.413
% Cancer	5.0	0.0	0.589
% Other chronic diseases	97.5	93.5	0.412
% Chronic use of drugs***	92.5	100	0.119

*calculated as percentages of vaccinated people considering the 71 subjects with available data for previous vaccinations.

**it was possible for each subject to have more than one disease.

***drugs most frequently used were antihypertensive/inotropic drugs and benzodiazepines.

### Immunogenicity of 2011–2012 IM-MF59 and ID influenza vaccines: hemagglutination inhibiting (HI) antibody response to the three vaccine antigens and persistence

Vaccine immunogenicity was evaluated by comparing HI titers in blood samples collected before and 30 days after vaccination and persistence of immune response by considering titers at 6 months of vaccination. The results are reported as protection rate (numbers of volunteers showing HI titers ≥40, considered to be associated with protection from influenza infection [15]), geometric mean titers (GMT), mean fold increase (MFI) of GMT (ratio of post-immunization to pre-immunization titers), seroconversion rate (subjects with a fourfold or greater increase in titer in pre-vaccination seropositive subjects or from <10 to ≥40 in seronegative volunteers).

As reported in Table 2, the pre-vaccination seroprotection rate and the values of GMT were similar in the IM-MF59 and ID groups and a significant increase in these values was observed 1 month after vaccination in both vaccine groups against the A(H3N2) and A(H1N1) vaccine components. The increases against the B vaccine antigen were not significant, except for GMT values in the ID group. HI titers at 6 months of vaccination decreased, as compared with those found at 1 month in both groups.

**Table 2:** HI antibody response against the three 2011-2012 influenza vaccine antigens 1 and 6 months after IM-MF59 or ID vaccination

**Vaccine component**	**Group (N)**	**Seroprotection rate (%)**				**GMT**		**MFI**		**Seroconversion rate (%)**	
		**[95% C.I.]**			**[95% C.I.]**			**[95% C.I.]**		**[95% C.I.]**	
		**Pre-vacc.**	**1 month**	**6 months**	**Pre-vacc.**	**1 month**	**6 months**	**1 month**	**6 months**	**1 month**	**6 months**
A/Perth/16/09 (H3N2)	IM-MF59	50.0	87.5**	66.5	25.5	83.7**	53.8*	3.3	2.1	47.5	17.5
	(40)	[37.3-62.6]	[76.4-93.8]	[52.0-76.1]	[16.3-40.1]	[44.8-156.2]	[27.3-106.2]	[1.9-5.7]	[1.1-4.1]	[35.1-60.2]	[9.8-29.4]
	ID	50.0	92.5**	67.5	28.3	131.3**	69.1	4.6	2.4	60.0	30.0
	(40)	[37.3-62.6]	[82.6-97.0]	[54.5-78.2]	[14.7-54.5]	[72.3-238.5]	[33.2-144.0]	[2.8-7.8]	[1.3-4.4]	[47.0-71.7]	[19.6-42.9]
A/California/7/09 (H1N1)	IM-MF59	25.0	72.5**	40.0	15.0	55.0**	22.0	3.7	1.5	50.0	7.5
	(40)	[15.5-37.6]	[59.7-82.4]	[28.3-53.0]	[9.2-24.5]	[31.9-94.8]	[13.0-37.3]	[2.4-5.6]	[1.1-2.0]	[37.3-62.6]	[3.0-17.4]
	ID	32.5	70.0**	50.0	15.8	61.0**	29.7	3.8	1.9	42.5	17.5
	(40)	[21.7-45.5]	[57.1-80.3]	[37.3-62.6]	[9.0-28.0]	[31.4-118.4]	[17.0-52.5]	[2.2-6.8]	[1.2-2.8]	[30.5-55.5]	[9.8-29.4]
B/Brisbane/60/08	IM-MF59	55.0	75.0	57.5	31.5	50.8	31.5	1.6	1.0	10.0	0.0
	(40)	[42.1-67.2]	[62.4-84.4]	[44.5 -69.5]	[18.8–52.7]	[29.6-87.0]	[18.5-53.5]	[1.3-2.0]	[0.9-1.1]	[4.6-20.5]	[0.0-6.3]
	ID	40.0	75.0	60.0	20.0	62.9*	35.9	3.2	1.8	40.0 ^$$^	17.5 ^$$^
	(40)	[28.3-53.0]	[62.4-84.4]	[47.0-71.7]	[12.7-32.0]	[35.6-114.7]	[20.7-62.2]	[1.7-6.0]	[1.0-3.1]	[28.3-53.0]	[9.8-29.4]

*p < 0.05 and **p < 0.01 comparing pre and post-vaccination data.

^$$^p < 0.01 comparing the two vaccine groups.

No significant differences were observed across vaccine groups against the A(H3N2) and A(H1N1) vaccine antigens 1 and 6 months after vaccination. On the contrary, serocoversion rates against the B antigen were higher, both at 1 (40.0% vs. 10.0%, *p* < 0.01) and 6 (17.5% vs. 0.0%, *p* < 0.01) months, in the ID group as compared with the IM-MF59 group.

The serological results observed 1 month post vaccination were also evaluated according to the Committee for Medicinal Products for Human Use (CHMP) criteria for approval of influenza vaccines in the elderly, which require that at least one of the following criteria must be met, protection rate ≥60%, MFI of GMT ≥2 and seroconversion rate ≥30% [16]. Although the pre-requisite of at least 50 persons per group was not met (the two groups examined were of only 40 people) and although there are some controversies on the identification of a single threshold (HI titer ≥40) for defining protection [17], all three CHMP criteria were met by both vaccines against A(H3N2) and A(H1N1) viruses and by ID vaccine against the B virus. IM-MF59 vaccine failed MFI and seroconversion criteria against B virus.

**Figure 1:**
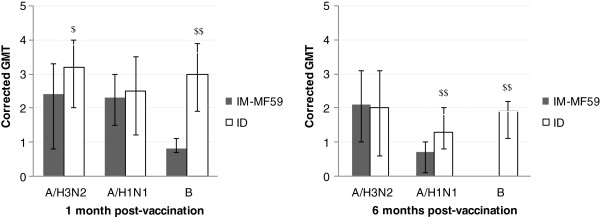
**One and 6 months post-vaccination GMT against the three 2011-12 influenza vaccine components adjusted according to pre-vaccination titers.** $: p < 0.05; $$: p < 0.01 comparing the two vaccine groups.

In order to have more comparable data, post-vaccination GMT values were adjusted for pre-vaccination status (Figure 1), as suggested by Beyer et al. [18]. One month after vaccination not only, as previously found, the responses against B virus (3.0 vs. 0.8, *p* < 0.01), but also against A(H3N2) virus (3.2 vs. 2.4, *p* < 0.05) were significantly higher in the ID group as compared with the IM-MF59 group. Six months after vaccination statistically higher values were found in the ID group against A(H1N1) (1.3 vs. 0.7, *p* < 0.01) and B (1.9 vs. 0.0, *p* < 0.01) viruses.

### Local influenza virus circulation and HI antibody response induced by IM-MF59 and ID influenza vaccines against epidemic drifted A(H3N2) viruses circulating in the 2011–2012 Winter season

**Figure 2:**
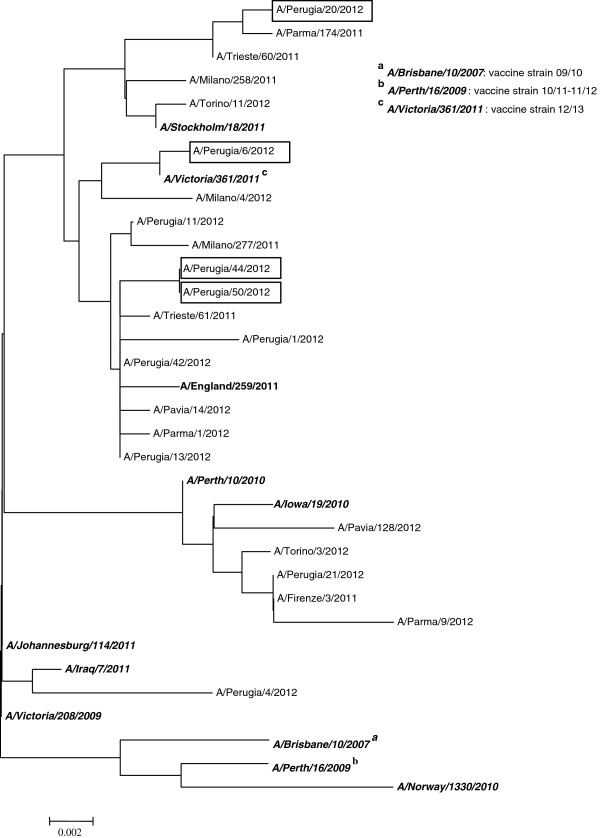
**Phylogenetic analysis of drifted A (H3N2) viruses circulating in the 2011–2012 winter.** The phylogenetic tree represents the analysis of the HA1 nucleotide sequences of the HA gene of different influenza A(H3N2) viruses isolated in Italy in the Winter season 2011–2012. The four epidemic drifted A(H3N2) viruses isolated in Umbria (A/Perugia/06/12; A/Perugia/20/12; A/Perugia/44/12; A/Perugia/50/12) were used as antigens for studying heterologous antibody responses induced by IM-MF59 and ID influenza vaccine.

During the 2011–2012 Winter season in our laboratory, the regional reference laboratory for Umbria of INFLUNET (Italian Surveillance Influenza Network), a total of 61 influenza viruses were identified on examining 91 throat swabs by cultivation in Madin-Darby canine kidney (MDCK) cells and/or Real-Time Reverse-Transcriptase-Polymerase-Chain-Reaction (RT-PCR). Swabs were collected from people with influenza-like illness (ILI) living in the area where the two nursing homes were located. Most of the viruses isolated not only in Umbria (53/61, i.e. 87%), but also in other parts of Italy, were A(H3N2) viruses, presenting antigenic and genetic patterns different from the A(H3N2) component of the 2011–2012 influenza vaccine [19]. The results obtained analyzing the nucleotide sequence of the HA1 domain of the hemagglutinin (HA) gene of a selected number of these A(H3N2) viruses, encompassing four viruses isolated in Umbria, are reported in Figure 2. The data show a high genetic affinity of these four viruses with the recent circulating viruses belonging in the A/Victoria/208/2009 clade, different from the clade of the 2011–2012 A(H3N2) vaccine component (A/Perth/16/2009 clade). Because of the importance of documenting the ability of the influenza vaccine to induce heterologous immune responses, we examined the induction of HI antibody responses against those four drifted A(H3N2) viruses following immunization with the two different enhanced 2011–2012 influenza vaccines. The results obtained, as reported in Table 3, show that, in most instances, pre-vaccination titers were comparable in the two vaccinated groups (except for seroprotection rate in the ID group, higher than in the IM-MF59 group against A/Perugia/44/12, p < 0.05) and in the same range as those found against the vaccine A(H3N2) virus (Table 2). Seroprotection rates ranged between 25.0% and 65.0%, as compared to 50.0% and 50.0% and GMT values ranged, indeed, between 15.4 and 33.7, as compared with 25.5 and 28.3 against A(H3N2) vaccine antigen. One month after vaccination, significant increases were observed both in seroprotection and in GMT values, with the exception of seroprotection rate against the A/Perugia/06/12 virus. On comparing the two vaccinated groups, significantly higher values were found in the ID group as compared with the IM-MF59 group against A/Perugia/20/12 and A/Perugia/44/12 viruses if considering the values of seroprotection (72.5% vs. 47.5%, p < 0.05, 87.5% vs. 67.5% p < 0.05, respectively, Table 3) and against A/Perugia/06/12 on considering the values of GMT corrected for pre-vaccination status (2.5 vs. 1.9, *p* < 0.01, Table 3). Moreover, the post-vaccination values tended to be lower as compared to those observed against the A(H3N2) vaccine component. On comparing circulating (Table 3) with vaccine A(H3N2) (Table 2) viruses, the GMT values ranged, respectively, from 35.4 to 75.9 and from 83.7 to 131.3, the MFI of GMT from 2.0 to 2.5 and from 3.3 to 4.6, and the percentage of seroconversions from 17.5% to 37.5% and from 47.5% to 60.0%.

**Table 3:** HI antibody response against four drifted epidemic A(H3N2) viruses circulating in Umbria (A/Perugia/06/12; A/Perugia/20/12; A/Perugia/44/12; A/Perugia/50/12) after IM-MF59 or ID influenza vaccine

**Epidemic virus**	**Group (N.)**	**Seroprotection rate (%)**		**GMT**		**MFI**	**GMT corrected #**	**Seroconversion rate (%)**
		**[95% C.I.]**		**[95% C.I.]**		**[95% C.I.]**	**[95% C.I.]**	**[95% C.I.]**
		**Pre-vacc.**	**1 month**	**Pre-vacc.**	**1 month**	**1 month**	**1 month**	**1 month**
A/Perugia/06/12	IM-MF59	32.5	70.0**	20.7	43.6**	2.1	1.9	27.5
	(40)	[21.7-45.5]	[57.1-80.3]	[14.4-29.7]	[30.8-61.8]	[1.5-2.9]	[1.4-2.4]	[17.6-40.3]
	ID	45.0	82.5	24.2	58.6*	2.4	2.5^$$^	35.0
	(40)	[32.8-57.9]	[70.6-90.2]	[16.7-35.0]	[41.1-83.5]	[1.7-3.5]	[1.6-3.1]	[23.9-48.0]
A/Perugia/20/12	IM-MF59	25.0	47.5*	15.4	35.4**	2.3	1.9	25.0
	(40)	[15.6-37.6]	[35.1-60.3]	[10.0-24.1]	[23.3-53.3]	[1.6-3.4]	[1.0-2.4]	[15.6-37.6]
	ID	37.5	72.5**^$^	18.0	38.0**	2.1	1.9	17.5
	(40)	[26.1-50.5]	[59.7-82.4]	[12.1-26.9]	[26.6-54.3]	[1.5-3.0]	[1.4-2.4]	[9.8-29.4]
A/Perugia/44/12	IM-MF59	32.5	67.5**	20.0	50.1**	2.5	2.1	37.5
	(40)	[21.7-45.5]	[54.5-78.3]	[13.2-30.3]	[32.0-78.5]	[1.7-3.8]	[1.2-3.0]	[26.1-50.5]
	ID	55.0 ^$^	87.5**^$^	28.3	56.6**	2.0	2.6	25.0
	(40)	[42.1-67.2]	[76.4-93.8]	[18.6-42.9]	[41.2-77.6]	[1.4-2.9]	[2.0-3.0]	[15.6-37.6]
A/Perugia/50/12	IM-MF59	50.0	82.5**	26.9	61.7**	2.3	2.6	25.0
	(40)	[37.4-72.6]	[70.6-90.2]	[16.9-42.7]	[42.1-90.3]	[1.5-3.5]	[1.7-3.1]	[15.6-37.6]
	ID	65.0	90.0**	33.7	75.9**	2.3	2.4	20.0
	(40)	[52.0-76.1]	[79.5-59.4]	[21.2-53.4]	[50.6-113.9]	[1.5-3.3]	[1.8-2.8]	[11.6-32.2]

*p < 0.05 and **p < 0.01 comparing pre and post-vaccination data.

^$^p < 0.05 ^$$^p < 0.01 comparing the two vaccine groups.

^#^post-vaccination GMT values corrected for pre-vaccination status.

As reported in Table 3, of the three CHMP criteria, the seroprotection rate and the MFI of GMT were always met, with the exception of the seroprotection rate against A/Perugia/20/12 antigen (47.5%) after IM-MF59. The seroconversion rate was in most instances lower than 30.0%, except for IM-MF59 group against A/Perugia/44/12 (37.5%) and for ID group against A/Perugia/06/12 (35.0%).

## Discussion

This study describes the immunogenicity and the ability to prevent influenza infection of two seasonal trivalent influenza enhanced vaccines, commercially available during Winter 2011–2012, characterized by the prevalent circulation of drifted A(H3N2) influenza viruses. The two vaccines, Fluad® and Intanza® 15mcg, meant to address the challenge of immunosenescence using different approaches (MF59 adjuvant and intradermal route of administration) were administered to 80 elderly volunteers (40 for vaccine group) living in two nursing homes.

The data obtained, examining the responses against the three vaccine antigens (Table 2), are in accordance with previous reports demonstrating the ability of the two potentiated vaccines, IM-MF59 [8,12] and ID [9-12], to elicit antibody responses in elderly volunteers. One month after vaccination significant increases in HI antibody titers were observed against A(H3N2) and A(H1N1) vaccine strains, whereas the responses against the B vaccine antigen, as previously reported for traditional inactivated [20,21] and potentiated [9,22] influenza vaccines, were more limited.

A direct comparison of the HI antibody responses induced by the same two potentiated vaccines (IM-MF59 and ID) in elderly people was previously reported by Van Damme [23] for the 2007–2008 Winter and by Scheifele et al. [12] for the 2011–2012 Winter season, the same as the one we examined. Since Van Damme et al. [23] reported the seroprotection and seroconversion data as a figure, only GMT values and the fulfillment of CHMP parameters could be compared. The results of Van Damme et al. [23] and of Scheifele et al. [12] differ under some respects from ours. Considering the responses against the two A vaccine strains, post-vaccination GMT titers against the A(H3N2) strain were higher in the IM-MF59 as compared with the ID group both in Van Damme et al. [23] and Scheifele et al. [12], whereas our data did not evidence differences (Table 2). Indeed we found that the GMT corrected for pre-vaccination status were even higher in the ID vs. IM-MF59 group (Figure 2). In accordance with Van Damme et al. [23] we observed similar responses against the A(H1N1) strain, whereas Scheifele et al. [12] found higher post-vaccination GMT titers in volunteers vaccinated with IM-MF59 as compared with ID group. However the differences, although statistically significant, were marginal and the three CHMP requirements were always reached against the two A vaccine strains [12].

Examining the post-vaccination GMT values against the B strain, the results obtained by Scheifele et al. [12] could not be evaluated because of the high baseline antibody values precluding meaningful response assessment. Similar post-vaccination GMT were reported by Van Damme et al. [23] in the two groups, whereas our data (Table 2), evidenced a tendency for a higher immunogenicity in the ID group compared with IM-MF59, especially after GMT adjustment for pre-vaccination titers (Figure 2). Only one CHMP requirement was reached in the two vaccine groups examined by Van Damme et al. [23] and in our IM-MF59 group, whereas all three requirements were met in our ID group.

Many different explanations can account for these differences. All the volunteers examined were 65 years or more, however the mean age of our population was higher (over 80 years) as compared with the mean age of the other two studies (lower than 80 years). We examined prevalently frail elderly people living in nursing homes, whereas volunteers studied by Scheifele et al. [12] were in most instances not frail and not living in care facilities. Moreover, the number of volunteers we studied was very limited (80 people) as compared with Van Damme et al. (795 participants) [23] and Scheifele et al. (911 participants) [12].

The potential variability in the immunogenicity of the injected influenza strains might have influenced the results obtained since all the three antigenic strains of the 2011–2012 Winter season, studied by us and Scheifele et al. [12], were updated as compared with those of 2007–2008 vaccine examined by Van Damme et al. [23]. Previous contact with influenza virus due to natural infection or vaccination might also be considered. A high percentage of the people of the three trials received influenza vaccine in the previous years. However, the very high antibody titers against the B strain found by Scheifele et al. [12], seem to suggest a possible different natural circulation of influenza viruses in the countries where the three studies were performed. Moreover, the possible contribution of the use of ether-treated B virus in the HI tests performed by Scheifele et al. [12] needs to be considered.

The other aspect examined by us and by Scheifele et al. [12] was the persistence of the vaccine induced antibody responses in the longer term, i.e. 6 months after vaccination. In accordance with Scheifele et al. [12] we found that HI antibody titers decreased against all the three vaccine strains in both vaccine groups 6 months after vaccination (Table 2); the HI titers evaluated by Scheifele et al. [12] as seroprotection against the two A strains did not differ between the two vaccine groups in contrast with the results observed shortly after vaccination. Our results evidenced that the responses found in people vaccinated with ID vaccine tended to be slightly higher as compared with IM-MF59 group especially if MFI of GMT and seroconversions rates are taken in account.

Further considerations derive from the data obtained on investigating the ability of the two vaccines to induce cross-reactive antibodies against four epidemic A(H3N2) strains circulating in the Winter 2011–2012 and found to be closely genetically correlated to the A/Victoria/208/2009 clade, different from the A/Perth/16/2009 clade (vaccine strain) (Figure 2). For the first time, the two potentiated vaccines were directly compared and the results confirm previous data demonstrating the ability of MF59-adjuvanted and intradermal vaccines [14] to elicit cross-reactive antibodies against heterologous or circulating viruses in elderly people. Both IM-MF59 and ID vaccines induced favorable immune responses against the four A(H3N2) circulating influenza viruses examined and at least two (seroprotection rate and MFI of GMT) of the CHMP criteria were met (Table 3). No substantial differences were found between the two vaccine groups, although HI titers were somewhat higher in the ID group. However, the post-vaccination values against the four circulating viruses were substantially poorer than those against the homologous A(H3N2) virus. In accordance with these results, suggesting that the drifted circulating strains examined may have different antigenic patterns with possible impact on vaccine immunogenicity, the A/Perth/16/2009 vaccine strain was replaced for the 2012–2013 Winter by A/Victoria/361/2011, belonging to the A/Victoria/208/2009 clade [24].

## Conclusions

In conclusion, this study, although limited in size, confirmed that the use of MF59 adjuvant and intradermal vaccination appear to be appropriate strategies to address the challenge of declining immune responsiveness in the elderly after influenza vaccination. Both IM-MF59 and ID influenza vaccines for the 2011–2012 Winter season were found to induce significant antibody responses against the three vaccine antigens, although the responses against the B antigen and the persistence of antibodies 6 months after vaccination tended to be higher in subjects vaccinated with ID than in individuals receiving IM-MF59 vaccine. Moreover, the two vaccines induced immune responses against drifted circulating influenza A(H3N2) viruses, although to a lesser extent as compared with A(H3N2) vaccine antigen.

Since a systematic meta-analysis for IM-MF59 versus ID vaccine is not available, these results can be considered preliminary, awaiting more extensive examination and systematic evidence.

## Methods

### Study population and vaccination

The study included a total of 80 elderly people living in two nursing homes located in Umbria (Italy) immunized with a single dose of trivalent influenza vaccine in November 2011. Two commercialized vaccines were freely offered for the Winter season 2011–2012 by the Public Health Authorities of Umbria (Italy) to the high risk group of elderly people: intramuscular MF59-adjuvanted (Fluad®, Novartis Vaccines, Italy) (IM-MF59) and intradermal (Intanza® 15 mcg, Sanofi-Pasteur MSD, France) (ID) influenza vaccine. The two vaccines contained 15 mcg of A/Perth/16/09 (H3N2), A/California/7/09 (H1N1) and B/Brisbane/60/08, respectively in 0.5 ml (IM-MF59) or 0.1 ml (ID). After obtaining informed consent, subjects were randomly assigned to receive in the deltoid region one dose of intradermal Intanza® 15 mcg (ID) or of intramuscular Fluad® (IM-MF59). Forty volunteers were immunized with IM-MF59 and 40 with ID influenza vaccine. Serum samples were examined for each volunteer before and approximately 1 and 6 months after vaccination. The study was conducted in accordance with all relevant regulations and with ethical standards set out in the Helsinki Declaration and Good Practice Guidelines and since both vaccines were assigned to the two nursing homes for the vaccination of elderly residents within the annual influenza vaccination campaign and sera were leftover sera from samples collected for clinical routine controls, the study did not need to be registered as a formal trial. Demographic, current medical conditions, prescribed medications, and previous influenza vaccination data were obtained from each subject at the time of vaccination.

### HI *antibody assay and vaccine immunogenicity*

Serum samples taken from the same subject and frozen at −20°C were tested simultaneously for HI antibodies titers against different influenza antigens. HI titers were determined by a standard microtiter method using 0.5% turkey erythrocytes. All sera were treated with receptor-destroying enzyme and heat-inactivated at 56°C for 30 min to remove non-specific inhibitors [25]. To eliminate any subjective bias, HI titers determinations were determined in a blind fashion, i.e. with the tester unaware of which treatment the donor had received.

### Viruses

The antibody responses were evaluated against the three egg-grown vaccine strains and against four circulating A(H3N2) field viruses (A/Perugia/06/12, A/Perugia/20/12, A/Perugia/44/12 and A/Perugia/50/12) isolated examining throat swabs (by culturing in MDCK cells and by RT-PCR) from people with ILI living in Umbria, Italy. Viruses were genetically characterized by sequencing the complete HA1 domain of the HA gene with specific primers (deposited in Gisaid; A/Perugia/06/12 [EPI:438358], A/Perugia/20/12 [EPI:438360], A/Perugia/44/12 [EPI:392313] and A/Perugia/50/12 [EPI:392315]). PCR products were amplified as previously reported [26] and purified using QIAquick PCR purification kit (Qiagen). Nucleotide sequences were obtained with the Big Dye Terminator Cycle Sequencing v1.1 ready Reaction kit using an ABI PRISM 310 sequencer (Applied Biosystems) and were aligned by using ClustalW 105 program (EMBL-EBI, European Bioinformatics Institute). Phylogenetic analysis was performed by using version 3.1 of the MEGA software package [27]. The Kimura-2-distance method and the Neighbor-Joining algorithm were used for the phylogenetic tree reconstruction.

### Statistical analysis

Differences between groups and results obtained using vaccine and epidemic antigens were analyzed by Student’s *t* test for continuous statistics (GMT), and by chi-square test for qualitative statistics (protection, seroconversion rate and clinical and demographic characteristics). HI titers were also transformed into binary logarithms, corrected for pre-vaccination status as described by Beyer et al. [18] and expressed as median titers, with the corresponding 25–75° inter-quartile range. Comparisons of corrected post-vaccination titers were analyzed by Wilcoxon test.

## Abbreviations

CHMP: Committee for medicinal products for human use; GMT: Geometric mean titer; HA: Hemagglutinin; HI: Hemagglutination inhibiting; ID: Intradermal trivalent influenza vaccine; ILI: Influenza like illness; IM-MF59: MF59-ajuvanted trivalent intramuscular influenza vaccine; MFI: Mean fold increase.

## Competing interest

The authors declare that they have no conflict of interest.

## Authors’ contributions

BC and MB participated in the design of the study, carried out immunological assays and performed the statistical analysis; ADM and ID carried out genetically characterizations of the viruses; AMI conceived of the study and draft the manuscript. All authors read and approved the final manuscript.
